# A Fast and Validated Reversed-Phase HPLC Method for Simultaneous Determination of Simvastatin, Atorvastatin, Telmisartan and Irbesartan in Bulk Drugs and Tablet Formulations

**DOI:** 10.3390/scipharm86010001

**Published:** 2017-12-19

**Authors:** Hassan A. Alhazmi, Ahmed M. Alnami, Mohammed A. A. Arishi, Raad K. Alameer, Mohammed Al Bratty, Zia ur Rehman, Sadique A. Javed, Ismail A. Arbab

**Affiliations:** 1 Department of Pharmaceutical Chemistry, College of Pharmacy, Jazan University, Jazan 45142, Saudi Arabia; ahmed.alnoaimi@gmail.com (A.M.A.); sargo_4@hotmail.com (M.A.A.A.); raadalameer@hotmail.com (R.K.A.); malbratty@jazanu.edu.sa (M.A.B.); ziaurrehman1@gmail.com (Z.u.R.); sajaved@jazanu.edu.sa (S.A.J.); ismailupm2011@gmail.com (I.A.A.); 2 Department of Pharmacy, IBMER, Mangalayatan University, 33rd Milestone, Beswan 202145, Aligarh, India; 3 Department of Chemistry, Faculty of Education, University of West Kordufan, El Nahud, West Kordufan State, Sudan

**Keywords:** reversed-phase HPLC, atorvastatin, simvastatin, telmisartan, irbesartan

## Abstract

The aim of this study was to develop and validate a fast and simple reversed-phase HPLC method for simultaneous determination of four cardiovascular agents—atorvastatin, simvastatin, telmisartan and irbesartan in bulk drugs and tablet oral dosage forms. The chromatographic separation was accomplished by using Symmetry C18 column (75 mm × 4.6 mm; 3.5 μ) with a mobile phase consisting of ammonium acetate buffer (10 mM; pH 4.0) and acetonitrile in a ratio 40:60 *v*/*v*. Flow rate was maintained at 1 mL/min up to 3.5 min, and then suddenly changed to 2 mL/min till the end of the run (7.5 min). The data was acquired using ultraviolet detector monitored at 220 nm. The method was validated for linearity, precision, accuracy and specificity. The developed method has shown excellent linearity (R^2^ > 0.999) over the concentration range of 1–16 µg/mL. The limits of detection (LODs) and limits of quantification (LOQs) were in the range of 0.189–0.190 and 0.603–0.630 µg/mL, respectively. Inter-day and intra-day accuracy and precision data were recorded in the acceptable limits. The new method has successfully been applied for quantification of all four drugs in their tablet dosage forms with percent recovery within 100 ± 2%.

## 1. Introduction

**Figure 1:**
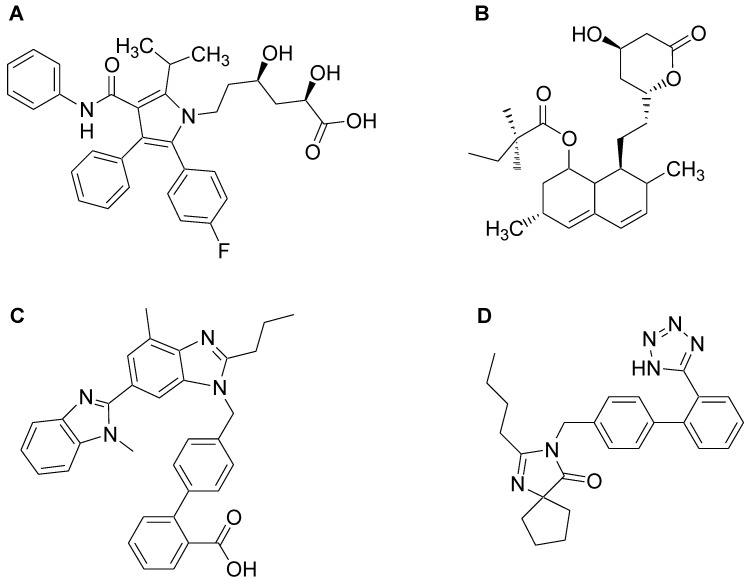
Chemical structures of (**A**) Atorvastatin (ATV); (**B**) Simvastatin (SMV); (**C**) Telmisartan (TLN) and (**D**) Irbesartan (IRB).

Atorvastatin (ATV) is chemically (3*R*,5*R*)-7-[2-(4-fluorophenyl)-3-phenyl-4-(phenylcarbamoyl)-5-propan-2-ylpyrrol-1-yl] 3,5-dihydroxyheptanoic acid (Figure 1A), while simvastatin (SMV) is [(1*S*,3*R*,7*S*,8*S*,8a*R*)-8-[2-[(2*R*,4*R*)-4-hydroxy-6-oxooxan-2-yl]ethyl]-3,7-dimethyl-1,2,3,7,8,8a-hexahydronaphthalen-1-yl]2,2-dimethylbutanoate (Figure 1B). Both ATV and SMV belong to the statins, which is a class of drugs that are used to lower blood cholesterol and triglyceride levels in the patients with cardiovascular complications and those at high risk for the development of atherosclerosis [1,2,3]. Statins exert their anti-hyperlipidemic effects through competitive inhibition of 3-hydroxy-3-methyl glutaryl coenzyme-A (HMG-CoA) reductase and hence inhibit a rate limiting step in biosynthesis of cholesterol. As a consequence, these drugs lower the risk of heart attack, stroke and related cardiac complications in individuals with coronary heart disease, type 2 diabetes and other cardiovascular conditions [4,5,6]. In addition to lipid lowering effect, these drugs also have been reported to possess anticancer [7,8], immunomodulatory [9], anti-inflammatory [9], anti-oxidant [10], anti-malarial [11] and antifungal [12] activities.

Telmisartan (TLN) is chemically, 2-[4-[[4-methyl-6-(1-methylbenzimidazol-2-yl)-2-propylbenzimidazol-1-yl]methyl]phenyl]benzoic acid (Figure 1C) and irbesartan (IRB) is 2-butyl-3-[[4-[2-(2H-tetrazol-5-yl)phenyl]phenyl]methyl]-1,3-diazaspiro[4.4]non-1-en-4-one (Figure 1D). TLN and IRB are selective non-peptide angiotensin II receptor antagonists, used for the management of hypertension. These drugs act by selectively blocking the AT1 receptors in the rennin-angiotensin system, and administered as single or combined with other anti-hypertensive agents [13].

Literature survey reveals that a number of analytical methods have been developed for the determination of atorvastatin in single as well as in combined dosage forms by UV-spectrophotometry [14,15,16,17] and reversed-phase high performance liquid chromatography (RP-HPLC) [18,19,20,21] methods. Similarly, various analytical methods for the quantification of simvastatin using UV-spectrophotometry [22,23,24] and HPLC [25,26,27] in single and multidrug formulations were reported in the literature. The methods based on high performance thin layer chromatography have also been reported for the determination of simvastatin in bulk drugs and marketed formulations [28]. Various methods are also available for the analysis of telmisartan in single dosage forms as well as in combinations with other drugs by UV-spectrophotometry [29,30,31] and liquid chromatographic techniques such as HPLC [32,33], HPTLC [34,35]and supercritical fluid chromatography [36]. Furthermore, several methods for the quantification of irbesartan in bulk drug and single as well as combined formulations have been reported by using UV-spectrophotometric [36,37,38,39] and HPLC [40,41] methods. Our study endeavors to develop and validate a reversed-phase HPLC method for simultaneous quantification of ATV, SMV, TLM and IRB in bulk drugs and their tablet dosage forms. To the extent of our knowledge, no analytical method based on reversed-phase HPLC have been reported so far, for the simultaneous estimation of the quaternary mixture containing ATV, SMV, TLM and IRB. Therefore, the current study was aimed to develop a rapid, simple and reproducible reversed-phase HPLC method for simultaneous quantification of the above cardiovascular drugs in bulk and tablet dosage forms and to validate the new method as per International Conference on Harmonization (ICH) method validation guidelines [42]. An attempt was made to choose a mobile phase that would be compatible with liquid chromatography-mass spectrometry, so that the developed method may be applicable for estimation of the test drugs in blood plasma samples during their pharmacokinetic monitoring. With the advent of combination therapy of anti-hyperlipidemic and anti-hypertensive agents, the proposed method will serve as a guide for their determination in API as well as in the formulations.

## 2. Materials and Methods

### 2.1. Chemicals and Reagents

The reference standards of atorvastatin, simvastatin, telmisartan and irbesartan were procured from Sigma Aldrich, Steinheim, Germany. Acetonitrile solvent (AR)-grade acetic acid, ammonium acetate, ammonium hydroxide and HPLC-grade acetonitrile were procured from Sigma Aldrich. Water (HPLC-grade) was prepared in our laboratory using Milli-Q water purification system (Millipore, Molsheim, France). Avapro^®^ (Sanofi Aventis, Bridgewater, NJ, USA), Atorva^®^ (Jazeera Pharmaceutical Industries, Riyadh, Saudi Arabia), Micardis^®^ (Boehringer Ingelhein, Ingelhein am Rhein, Germany) and Zocor^®^ (Merck Sharp & Dohme Corporation, Whitehouse Station, NJ, USA) tablets consisting 75 mg, 20 mg, 80 mg and 20 mg of IRB, ATV (as calcium trihydrate), TLM and SMV respectively were purchased from the local community pharmacy.

### 2.2. Instrumentation and Chromatographic Conditions

The analysis was carried out by using Waters HPLC system (Waters Breeze 1525, Etten-Leur, The Netherlands) equipped with binary pump (Waters 1525), autosampler (Waters 2707) and UV-Visible detector (Waters 2489). The system was monitored by using Waters Breeze 2 LC solution software. HPLC separation was carried out on a Symmetry C18 column (75 mm × 4.6 mm; 3.5 µm) maintained at ambient temperature with a mobile phase consisting of 10 mM ammonium acetate buffer (pH 4) and acetonitrile with ratio 40:60 *v*/*v*. A volume of 20 μL was injected and the flow rate was maintained at 1 mL/min up to 3.5 min, then suddenly increased to 2 mL/min up to the end and all the analytes were monitored at 220 nm, set as detection wavelength. The run time for all the analysis was 7.5 min. 0.77 g of ammonium acetate was accurately weighed and dissolved in 1 L of HPLC grade water. The pH of resulting solution was adjusted to 4.0 with the help of glacial acetic acid. The solution was then filtered through nylon filter (0.45 μ) to remove any suspended particles. Mobile phase was prepared by mixing ammonium acetate buffer and acetonitrile as an organic modifier in the ratio 40:60 *v*/*v* and then sonicated for 10 min to degas.

### 2.3. Preparation of Solutions

#### 2.3.1. Standard Stock Solutions

The standard stock solutions (1 mg/mL) of IRB, ATV and SMV were prepared by dissolving an accurately weighed 10 mg of each reference standards separately in 10 mL of methanol. The stock solution of TLM was prepared by dissolving 10 mg of telmisartan standard in a mixture of methanol and acetonitrile (60:40 *v*/*v*) in a 10-mL volumetric flask.

#### 2.3.2. Working Standard Solutions

The working standard solutions of IRB, ATV, TLM and SMV were obtained by diluting the respective standard stock solutions with a mixture of water and acetonitrile (1:1) to prepare calibration curve standards in the concentration range of 1–16 μg/mL.

#### 2.3.3. Sample Solutions

As the combined formulation containing all the tested actives was not available in the market, the individual marketed tablet formulations were purchased for the analysis. Ten tablets of each drug were crushed and a weight equivalent to 10 mg of ATV, SMV, TLM and IRB were transferred separately to 10 mL volumetric flasks and sonicated with methanol (for TLM, a mixture of methanol and acetonitrile, 60:40 *v*/*v* was taken) for 20 min. The volume of each mixture was made and filtered through 0.45 μm nylon syringe filter. The solutions were then diluted with a mixture of water and acetonitrile (1:1 *v*/*v*) to get the final concentration of 10 μg/mL of each drug component.

### 2.4. Method Validation

The validation of the newly developed reversed-phase HPLC method was performed as per ICH guidelines of Validation of Analytical Procedure: Q2 (R1) [42]. The parameters of validation such as system suitability, linearity, limit of detection (LOD), limit of quantification (LOQ), precision, specificity, accuracy, recovery and solution stability were addressed.

#### 2.4.1. System Suitability

To ensure the system performance, the suitability of the system was determined before and during analysis of unknown samples. Number of theoretical plates, retention time, peak separation (resolution), tailing factor and percent relative standard deviation (%RSD) of area were assessed by injecting six replicates of standard solution. The system was considered to be suitable for the analysis when number of theoretical plates more than 2000, the tailing factor less than 2.0 and the %RSD of the area of six replicate injections less than 2.0% were observed.

#### 2.4.2. Linearity

The assessment of the detector response linearity was performed by analyzing a series of different concentrations of working standards (mixture of all analytes). The concentration range was selected at five point levels, viz. 1, 2, 4, 8 and 16 µg/mL. All the solutions were injected into the system in six replicates and the corresponding area of each peak was recorded. Furthermore, the calibration curve was plotted by using the area of peaks of each component versus respective concentrations. The linearity of the method was calculated by regression analysis. A HPLC method is considered to be linear if it shows a correlation coefficient (*R*^2^) > 0.999.

#### 2.4.3. Precision and Accuracy

The newly developed method was evaluated for its precision and accuracy by the analysis of the quality control samples containing all the analytes at three different concentrations, i.e., low quality control (2 μg/mL, LQC), medium quality control (8 μg/mL, MQC) and high-quality control (16 μg/mL, HQC) levels. Intra-day precision and accuracy was assessed by analyzing six replicates of the sample and standard solutions in the same day at three different times, whereas inter-day precision and accuracy was established by performing same analysis over three successive days. The %RSD of the observed peak area of each component at all concentration levels and the % recoveries were calculated.

#### 2.4.4. Limit of Detection and Limit of Quantification

The LODs and LOQs for ATV, SMV, IRB and TLM were calculated by using the below mentioned Equations (1) and (2):

(1) $$LOD=\frac{3.3\times SD}{s}$$

(2) $$LOQ=\frac{10\times SD}{s}$$

where, *SD* represents the standard deviation corresponding to *Y*-intercept regression line and *s* denotes the slope of the calibration curve.

#### 2.4.5. Specificity

Specificity was carried out to determine the presence of interference due to co-eluting peaks (from blank) at the retention times of the analytes under investigation. To check the specificity of the proposed method, placebo solution was prepared in the same way as sample solution and injected in the HPLC system. Furthermore, the interference due to blank was detected by injecting the diluent separately. The chromatogram from the blank solution was compared with that of the sample solutions of bulk drugs as well as the tablet dosage forms.

#### 2.4.6. Solution Stability

The stability of the sample and standard solutions was assessed by storing at different conditions including; 25 °C (ambient condition) for 12 h (bench-top stability), 4 °C (refrigerator temperature) for 14 days and −20 °C for 30 days. At the end of each storage time the solutions were injected in the HPLC column and the results were compared with that of freshly prepared sample solutions. The stability analysis was performed using analytical solutions at MQC level.

## 3. Results and Discussion

### 3.1. Method Development and Optimization

The widespread use of lipid lowering drugs such as atorvastatin and simvastatin and anti-hypertensive agents such as telmisartan and irbesartan for the management of cardiovascular complications has stimulated our interest to develop a fast and simple analytical method for the simultaneous determination of the quaternary mixture of ATV, SMV, TLM and IRB in bulk and tablet formulations. The chromatographic parameters of the current method were optimized after various trials. The emphasis was laid on the selection of a volatile buffer, so that the method could easily be transferred to hyphenated techniques like LC-MS for bio-analysis of the tested drug products. Hence, ammonium acetate buffer which is compatible with LC-MS was selected for this study. In the optimization process, different buffer pH, mobile phase compositions and two different detector wavelengths were tried. Based on the ionization of ATV, SMV, TLM and IRB two pH levels, acidic (pH 4.0) and alkaline (pH 8.5) were selected. Mobile phase compositions of 50:50, 60:40, 70:30, 40:60 and 30:70 *v*/*v* of ammonium acetate buffer and acetonitrile were tried. Poor separation and peak symmetry of the tested analytes were observed when a mobile phase with alkaline ammonium acetate buffer was used. At the ratios of 60:40 and 70:30 *v*/*v* ammonium-acetate buffer (pH 8.5) and acetonitrile in the mobile phase, only two drugs were eluted with satisfactory resolution in about 20 minutes run time at flow rate equal to 1 mL/min.

**Figure 2:**
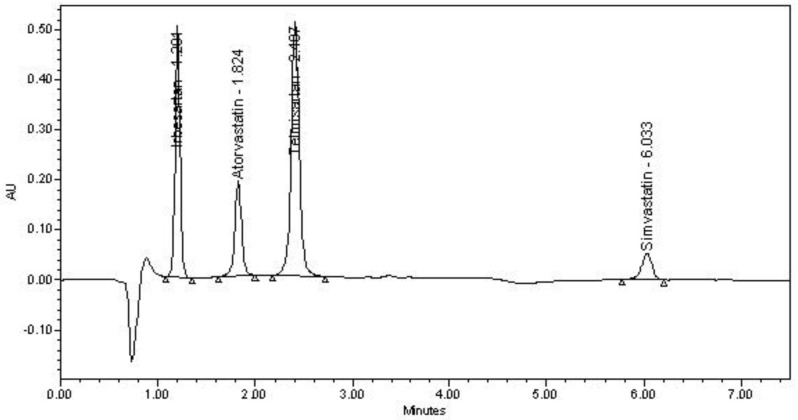
Chromatogram showing excellent separation between irbesartan, atorvastatin, telmisartan and simvastatin. *Conditions:* stationary phase, Symmetry C18 column; mobile phase, 10 mM ammonium acetate buffer (pH 4)–acetonitrile (40:60 *v*/*v*); flow rate, 1 mL/min up to 3.5 min then 2 mL/min; detection, UV 220 nm.

Different compositions of acidic ammonium-acetate buffer (pH 4.0) and acetonitrile in the mobile phase were tried. A high-quality separation and symmetric peak shape for all the tested analytes was achieved with mobile phase made up of 40:60 *v*/*v* ammonium-acetate buffer (pH 4.0) and acetonitrile at 1 mL/min flow rate. With this composition, SMV was eluted at 9.1 min, while the retention time for IRB, ATV and TLM were observed at 1.20, 1.82 and 2.40 min, respectively. The retention time of SMV was reduced by using sudden change in flow rate, in which the flow rate was kept 1 mL/min up to 3.5 min, and then increased to 2 mL/min until the end of the run. As a result, SMV was eluted at the retention time of about 6 min without affecting the retention times of other analytes and finally a runtime of 7.5 min was finalized for this analysis. Furthermore, the number of theoretical plates and tailing factor were also found to be in acceptable limits. The representative chromatogram of the finalized chromatographic conditions showing separation of peaks due to IRB, ATV, TLM and SMV is depicted in Figure 2.

### 3.2. Method Validation

The developed method was validated as per the criteria set by ICH guidelines. The validation was performed according to the parameters: linearity, precision and accuracy, system suitability, specificity and solution stability. The data obtained for each validation parameter were found well within the acceptance limits.

#### 3.2.1. System Suitability

The number of theoretical plates, resolution, tailing factor and %RSD of six replicate injections of the working standard solution were evaluated. The peaks due to all the analytes were found to be symmetrical as the observed tailing factors were less than 2 and the resolution between the drug peaks were satisfactory (>2). The observed system suitability parameters of the present method were within the acceptable limits set by US Food and Drug Administration (FDA) regulations [43], which indicate the suitability of the system. The system suitability data of the present method are summarized in Table 1.

#### 3.2.2. LOD and LOQ

The values of LOD in the developed method were 0.189, 0.190, 0.189 and 0.189 µg/mL for ATV, SMV, TLM and IRB respectively. The calculated LOQ values obtained were 0.603, 0.604, 0.603 and 0.630 µg/mL for ATV, SMV, TLM and IRB respectively.

#### 3.2.3. Specificity

The developed method was proven to have specificity as there was no significant interference from blank or placebo at the retention times of the analytes (ATV, SMV, TLM and IRB) observed. Furthermore, no carry-over effect was noticed throughout the analysis.

#### 3.2.4. Linearity

**Figure 3:**
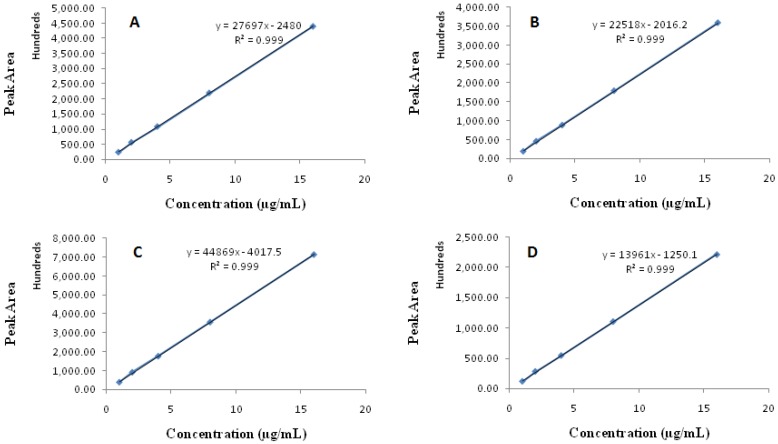
Calibration curve showing excellent linearity of the method: (**A**) Irbesartan; (**B**) Atorvastatin; (**C**) Telmisartan and (**D**) Simvastatin.

The linearity of the developed method was assessed by preparing the calibration plot between the area of the peaks versus concentrations of IRB, ATV, TLM and SMV over the range of 1–16 µg/mL (Figure 3). Linear calibration plots were obtained for all the tested drugs and the mean correlation coefficients (*R*^2^) for individual analytes was achieved to be >0.999, which suggest that the proposed method is linear.

#### 3.2.5. Precision and Accuracy

The precision and accuracy data for both inter- and intra-day analysis of IRB, ATV, TLM and SMV in the quality control samples at three levels are depicted in Table 2. The inter-day precision values ranged from 1.35–2.06%, 0.97–1.61%, 0.36–1.39% and 0.38–1.52% for ATV, SMV, TLM and IRB respectively, while the intra-day precision (%RSD of peak area) ranged from 1.39–2.10%, 0.46–1.41%, 1.27–1.58% and 0.38–1.89% for ATV, SMV, TLM and IRB respectively. Similarly, the recovery values for all the tested analytes were within range of 98–102%. The results of precision and accuracy were found to be within the acceptance limits and revealed that the new method is precise and accurate.

#### 3.2.6. Solution Stability

There was no stability related problem observed during the course of analysis under different conditions. The test and working standard solutions showed good stability at laboratory temperature for 12 h (average time of analysis), 4 °C (refrigerator temperature) for 14 days and −20 °C for 30 days. The stability of the analytical solutions was expressed as average percent recoveries, which were found to be in the range from 99.12–100.12%, 99.74–99.87%, 100.69–101.59% and 100.96–101.56% for ATV, SMV, TLM and IRB, respectively. The stability results in this study were found to be within the acceptable limits (±2%), which suggests that the sample and standard solutions can be evaluated under normal laboratory environment without any significant loss. The solution stability results are depicted in Table 3.

### 3.3. Application of the Developed Method in Determination of ATV, SMV, TLM and IRB in Tablet Dosage Forms

**Figure 4:**
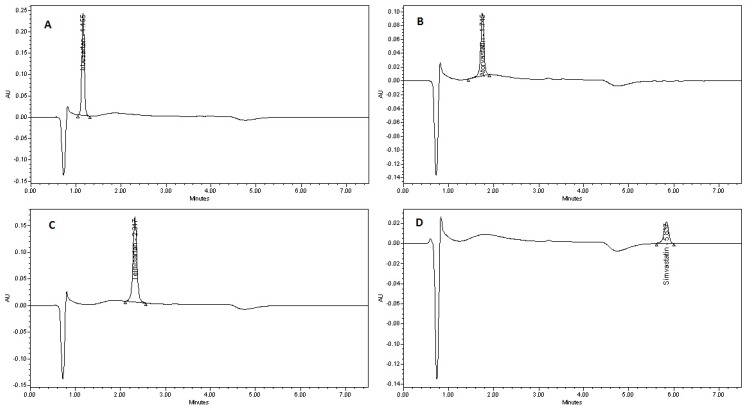
Representative chromatograms of individual analytes in tablet dosage forms. (**A**) Irbesartan; (**B**) Atorvastatin; (**C**) Telmisartan; and (**D**) Simvastatin. *Conditions*: stationary phase, Symmetry C18 column; mobile phase, 10 mM ammonium acetate buffer (pH 4)–acetonitrile (40:60 *v*/*v*); flow rate, 1 mL/min up to 3.5 min then 2 mL/min; detection, UV 220 nm.

The developed method was successfully applied for the quantification of IRB, ATV, TLM and SMV in their tablet formulations commercially available in the local market. Figure 4 shows the representative individual chromatograms of IRB, ATV, TLM and SMV in their tablet formulations. No variation in the runtime and other system suitability parameters were observed for all the component drugs. Furthermore, no interference from the placebo was noticed at the retention time of each analyte. The accuracy of this method in tablet formulation of each drug was determined by spiking method, where the recovery samples were prepared at 50%, 100% and 150% levels of the targeted concentration (10 μg/mL). At each concentration level the recovery samples were prepared and injected in triplicate to the HPLC system in six replicates. The percent recovery of the amount of each drug component at every concentration level was calculated by determining their contents from the respective chromatograms. The average percent recovery of each analyte was found to be within 100 ± 2%. The calculated average percent recoveries for tablet formulations has been shown in Table 4.

## 4. Conclusions

A fast and simple RP-HPLC method for the simultaneous quantification of atorvastatin, simvastatin, telmisartan and irbesartan has been successfully developed. The elution time for all the analytes was short (7.5 min) and found to have excellent peak shapes and acceptable system suitability parameters. The new method was validated as per ICH guidelines and it was found to be linear, precise, accurate and specific. All the analytes were stable in different storage conditions from laboratory temperature to −20 °C. The developed method was applied successfully for the quantification and detection of ATV, SMV, TLM and IRB in their tablet oral dosage forms. The adaptability of this method to tablet formulations was proven by its excellent performance in terms of specificity and recovery for each drug component in the tablet samples. Therefore, the method may be utilized for day to day quality control (QC) analysis of IRB, ATV, TLM and SMV in bulk drugs or in their tablet dosage forms. The major advantage of the current method is that it is less time consuming and the ammonium acetate buffer used in mobile phase is compatible with LC-MS.

## Figures and Tables

**Table 1:** Retention times, tailing factor, resolution, capacity factor and number of theoretical plate for atorvastatin (ATV), simvastatin (SMV), telmisartan (TLM) and Irbesartan (IRB) recorded by the developed HPLC method.

Parameters	IRB	ATV	TLM	SMV
Retention time (min)	1.20	1.82	2.40	6.03
USP Tailing factor	1.12	0.98	1.18	1.08
USP Resolution		5.92	4.21	20.63
USP Plate count	2701	3621	4299	15122
Percent RSD of peak area (*n* = 6)	0.41	0.82	0.36	0.89

USP: United States Pharmacopeia.

**Table 2:** Precision and accuracy data of intra-day and inter-day samples for the proposed HPLC method, as carried out at three quality control levels for ATV, SMV, TLM and IRB.

	Concentration Levels (µg/mL)	ATV	SMV	TLM	IRB
Intra-day Precision and Accuracy					
%RSD of peak area (Average % recovery)	2	2.10 (101.99)	1.41 (100.75)	1.39 (102.14)	1.89 (100.41)
	8	1.61 (100.21)	0.46 (100.30)	1.58 (100.26)	0.38 (100.75)
	16	1.39 (100.07)	1.30 (100.32)	1.265 (100.34)	1.07 (100.83)
Inter-day Precision and Accuracy					
%RSD of peak area (Average % recovery)	2	2.06 (101.95)	1.52 (102.78)	1.39 (102.02)	1.52 (102.64)
	8	1.60 (100.32)	0.97 (99.99)	0.69 (99.98)	0.38 (101.38)
	16	1.35 (100.10)	1.61 (101.34)	0.36 (100.80)	1.07 (101.20)

2, 8 and 16 μg/mL are low, medium and high-quality control sample concentrations, respectively; *n* = 3.

**Table 3:** Solution stability data at different storage conditions.

Analytes	Storage Conditions	Average %Recovery *
Atorvastatin	Normal laboratory temperature (25 °C) for 12 h	99.86
	Refrigerator temperature (4 °C) for 14 days	99.12
	−20 °C for 30 days	100.12
Simvastatin	Normal laboratory temperature (25 °C) for 12 h	99.74
	Refrigerator temperature (4 °C) for 14 days	99.87
	−20 °C for 30 days	99.84
Telmisartan	Normal laboratory temperature (25 °C) for 12 h	100.69
	Refrigerator temperature (4 °C) for 14 days	100.98
	−20 °C for 30 days	101.59
Irbesartan	Normal laboratory temperature (25 °C) for 12 h	100.96
	Refrigerator temperature (4 °C) for 14 days	101.56
	−20 °C for 30 days	101.26

* *n* = 3.

**Table 4:** Accuracy (recovery) data of ATV, SMV, TLM and IRB in tablet dosage forms.

Analytes	Recovery Sample Concentrations (μg/mL)	Percentage of Targeted Concentration	Amount of Drugs Recovered (μg/mL)	%Recovery ± %RSD *
Atorvastatin	5	50%	5.02	100.42 ± 0.57
	10	100%	10.18	101.81 ± 0.89
	15	150%	15.14	100.98 ± 0.73
Simvastatin	5	50%	4.94	98.86 ± 1.09
	10	100%	9.98	99.80 ± 1.09
	15	150%	14.95	99.67 ± 1.10
Telmisartan	5	50%	5.06	101.38 ± 1.58
	10	100%	10.12	101.20 ± 1.59
	15	150%	15.16	101.07 ± 1.59
Irbesartan	5	50%	5.07	101.50 ± 0.38
	10	100%	10.12	101.20 ± 0.38
	15	150%	15.15	101.05 ± 0.39

* *n* = 6.
